# Respiratory oscillations in alveolar oxygen tension measured in arterial blood

**DOI:** 10.1038/s41598-017-06975-6

**Published:** 2017-09-06

**Authors:** Federico Formenti, Nikhil Bommakanti, Rongsheng Chen, John N. Cronin, Hanne McPeak, Delphine Holopherne-Doran, Goran Hedenstierna, Clive E. W. Hahn, Anders Larsson, Andrew D. Farmery

**Affiliations:** 1 Nuffield Division of Anaesthetics, University of Oxford, Oxford, United Kingdom; 2 Faculty of Life Sciences and Medicine, King’s College London, London, United Kingdom; 3 School of Veterinary Sciences, University of Bristol, Langford, United Kingdom; 4 Department of Medical Sciences, University of Uppsala, Uppsala, Sweden; 5 Department of Surgical Sciences, University of Uppsala, Uppsala, Sweden

## Abstract

Arterial oxygen partial pressure can increase during inspiration and decrease during expiration in the presence of a variable shunt fraction, such as with cyclical atelectasis, but it is generally presumed to remain constant within a respiratory cycle in the healthy lung. We measured arterial oxygen partial pressure continuously with a fast intra-vascular sensor in the carotid artery of anaesthetized, mechanically ventilated pigs, without lung injury. Here we demonstrate that arterial oxygen partial pressure shows respiratory oscillations in the uninjured pig lung, in the absence of cyclical atelectasis (as determined with dynamic computed tomography), with oscillation amplitudes that exceeded 50 mmHg, depending on the conditions of mechanical ventilation. These arterial oxygen partial pressure respiratory oscillations can be modelled from a single alveolar compartment and a constant oxygen uptake, without the requirement for an increased shunt fraction during expiration. Our results are likely to contribute to the interpretation of arterial oxygen respiratory oscillations observed during mechanical ventilation in the acute respiratory distress syndrome.

## Introduction

In the healthy lung, arterial partial pressure of oxygen (PaO_2_) is thought to remain almost constant within the respiratory cycle during spontaneous breathing, via a physiological PO_2_ gradient between the alveoli and the pulmonary circulation^1^. Respiratory PaO_2_ oscillations smaller than 16 mmHg have been detected with slow response time sensors^2^ in animals with uninjured lungs when abnormally large tidal volumes (V_T_) greater than 20 ml kg^−1^ or even 30 ml kg^−1^ were delivered during mechanical ventilation^3, 4^.

In the injured lung, especially in animal models of the acute respiratory distress syndrome (ARDS), large PaO_2_ oscillations have been observed by means of fast-response oxygen sensing technology^5–12^. Here, PaO_2_ oscillations have been explained by variable within-breath shunt due to the repetitive opening and collapse of lung regions (cyclical atelectasis), causing PaO_2_ to increase during inspiration and decrease during expiration. This phenomenon may be used to personalize mechanical ventilation based on real-time, continuous PaO_2_ monitoring, where the appearance of an oscillating PaO_2_ signal may be a diagnostic biomarker for cyclical atelectasis.

A significant limitation of the above mentioned studies is that the PaO_2_ oscillations observed in animal models of ARDS mostly appeared in association with a high average V_T_ of 20 ml kg^−1^ ^7, 13^, 30 ml kg^−1^ ^6, 14^, or greater^10^. These large V_T_ were delivered in experiments without application of positive end-expiratory pressure (PEEP), and imposing elevated peak end-expiratory pressures in order to provoke cyclical atelectasis in the injured lung. Even with these large V_T_, PaO_2_ oscillations only between 3 and 22 mmHg were observed in the uninjured lung, at a RR lower than 10 breaths per minute, and mean PaO_2_ above 450 mmHg^6, 15^.

No study employing fast oxygen-sensing technology has explored the responses of the uninjured lung to mechanical ventilation at physiological PaO_2_ levels. Experimental evidence for this response to mechanical ventilation could also be useful to understand response of the lung to mechanical ventilation in the presence of injury.

We developed a fibre optic oxygen sensor^16^, optimized its response time and linearity^17–20^, tested it *in vitro* ^21^, showed it to be resistant to clotting, and demonstrated it to be capable of detecting rapid PaO_2_ changes in an animal model of ARDS^18, 34^. With this new technique to study gas exchange dynamically, we explore here PaO_2_ responses to mechanical ventilation in a porcine model.

We hypothesised that in apnoea or expiration PaO_2_ declines at a rate that is inversely proportional to lung volume, as had been modelled^22, 23^, and that this decline would be readily measurable in the uninjured pig lung during 20 s end-expiratory and end-inspiratory breath holds. We also hypothesised that, during tidal breathing, PaO_2_ oscillates dynamically in phase with lung volume in the uninjured lung, in the absence of cyclical atelectasis. This hypothesis led us to also explore the possibility that different ventilation strategies (pressure and volume control) and inspired-to-expired ratios could affect the mean PaO_2_ value, while airway pressures (end-expiratory and end-inspiratory), respiratory rate, or fraction of inspired oxygen (F_I_O_2_) were kept unvaried.

## Results

### PaO_2_ oscillates during tidal breathing with mechanical ventilation

**Figure 1:**
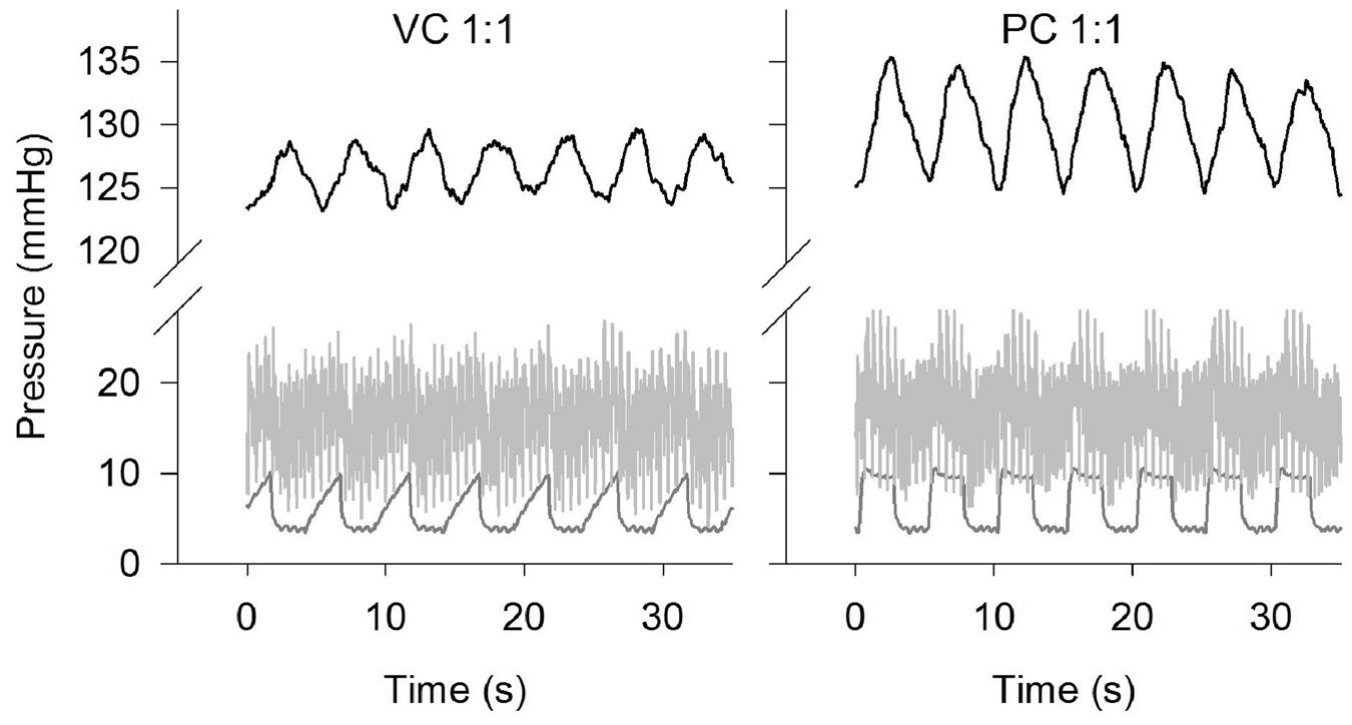
PaO_2_ oscillations in the uninjured, ventilated lung. Representative continuous measurements of PaO_2_ (top, black), pulmonary artery pressure (middle, light grey) and airway pressure (bottom, dark grey) are presented as a function of time. Ventilation was managed in volume control (left, VC) and pressure control (right, PC) mode. Inspired-to-expired ratio was 1:1, and respiratory rate was 12 breaths per minute.

Figure 1 illustrates the tidal nature of PaO_2_ as observed in an anesthetised pig with uninjured lungs during volume (VC) and pressure control (PC) mechanical ventilation. PaO_2_ oscillations with an amplitude of about 10 mmHg and the same frequency as breathing were observed at a RR of 12 breaths per minute, inspired-to-expired ratio (I:E) of 1:1, tidal volume (V_T_) of about 10 ml kg^−1^, and at mean PaO_2_ near 130 mmHg. The peak airway pressure was maintained below 13.6 cmH_2_O. The airway pressure remained lower than the mean pulmonary artery pressure throughout the experiment.

### The rate of PaO_2_ decline during breath holding depends on lung volume and metabolic rate, and is not associated with atelectasis in the uninjured lung

**Figure 2:**
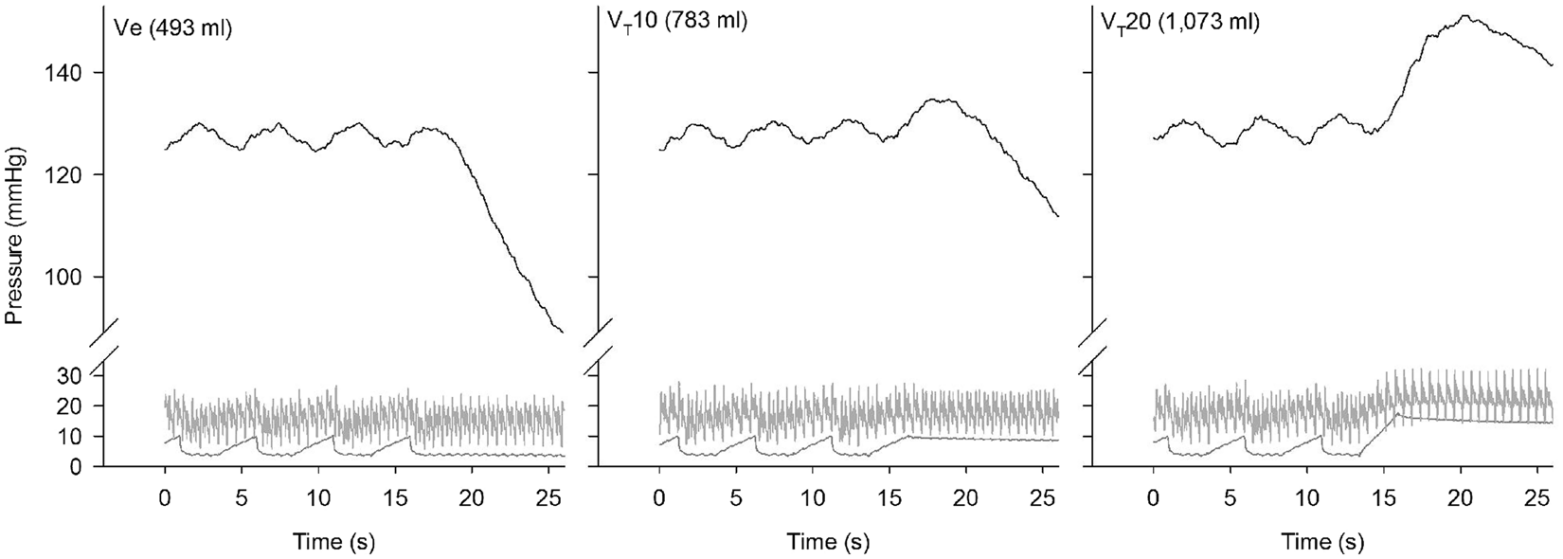
PaO_2_ changes during breath holding manoeuvres at three lung volumes. Representative continuous measurements of PaO_2_ (top, black), pulmonary artery pressure (middle, light grey), and airway pressure (bottom, dark grey) are presented as a function of time. Lung volumes measured for each breath hold are reported for Ve (end-expiratory breath hold with PEEP set at 3.7 mmHg [5 cmH2O], left), VT10 (end-inspiratory breath hold with VT of 10 ml kg^−1^, center), and VT20 (breath hold at the end of a large inspiration with VT of 20 ml kg^−1^, right). Ventilation was in volume control mode, inspired-to-expired ratio was 1:1, and respiratory rate was 12 breaths per minute. Associated CT images were captured at the beginning and end of the breath holding periods, and are presented in the supplementary information.

Figure 2 shows the rate of PaO_2_ decline over time during breath hold manoeuvres at end-expiratory volume (493 ml; left), end-inspiratory volume (783 ml; centre), and at the end of a large inspiration (1,073 ml; right). The figure illustrates the slower rate of decline observed at the larger lung volume, where also a transient increase in PaO_2_ was observed immediately following the large inspiration. There was good agreement between measurements of V_T_ recorded via the respiratory monitor (Datex Ultima) and estimates of the lung volume increases consequent upon inflation made by CT image analysis: the mean volume difference (i.e. bias) was −22 ml, with limits of agreement between −67 (95% CI −80 to −54) and 22 (95% CI 9 to 35). The SD of the differences was 23 ml (SE ± 3.7 ml) (Figure S1). Representative slice CT images recorded as soon as the breath hold manoeuvre began, and just before tidal breathing was restored are shown in Figure S2 for reference. Similarly, Figure S3 shows three-dimensional reconstructions of the lung obtained from CT images, highlighting regions associated with different Hounsfield Units ranges corresponding to well- or poorly-ventilated lung, and atelectasis.

Table 1 shows that the three lung volumes measured with CT during the breath hold manoeuvres were significantly different (*p* < 0.001), as were the associated rates of PaO_2_ decline (*p* < 0.01). The rates of PaO_2_ decline ranged from 6.9 ± 1.3 mmHg s^−1^ at end-expiratory lung volume to 3.4 ± 0.6 mmHg s^−1^ during the large inspiration (VT20). Table 1 also shows whole lung atelectatic mass fraction measured with CT as soon as the breath hold manoeuvre began and just before breathing was restored. There was no significant difference in the whole lung atelectatic mass fraction at the beginning and at the end of the breath hold manoeuvres [C.I. −1.4%–0.6%].

**Table 1:** Lung volumes estimated via CT at the beginning and at the end of breath-hold manoeuvres, associated whole lung atelectasis mass fraction and rates of PaO_2_ decline.

		End-expiration	End-inspiration	End large inspiration
Volume beginning (ml)	P1	681 ± 17	983 ± 31	1249 ± 66
	P2	546 ± 17	852 ± 90	1051 ± 84
Volume end (ml)	P1	624 ± 14	927 ± 31	1209 ± 69
	P2	495 ± 19	794 ± 94	1004 ± 88
Atelectasis beginning (%mass)	P1	12.6 ± 0.9	11.5 ± 0.7	11.3 ± 1.1
	P2	17.3 ± 0.4	16.1 ± 0.6	15.6 ± 0.5
Atelectasis end (%mass)	P1	13.4 ± 1.0	11.9 ± 0.9	11.1 ± 0.9
	P2	18.6 ± 0.7	16.4 ± 0.5	15.3 ± 0.4
PaO_2_ decline (mmHg s^−1^)		6.9 ± 1.3	4.4 ± 0.8	3.4 ± 0.6

Lung volumes and atelectasis were measured by CT in two animals only (P1 and P2); rate of PaO_2_ decline was measured in 8 animals. Values are average ± standard deviation.

### Respiratory timing affects the PaO_2_ mean value and the amplitude of its oscillations

**Figure 3:**
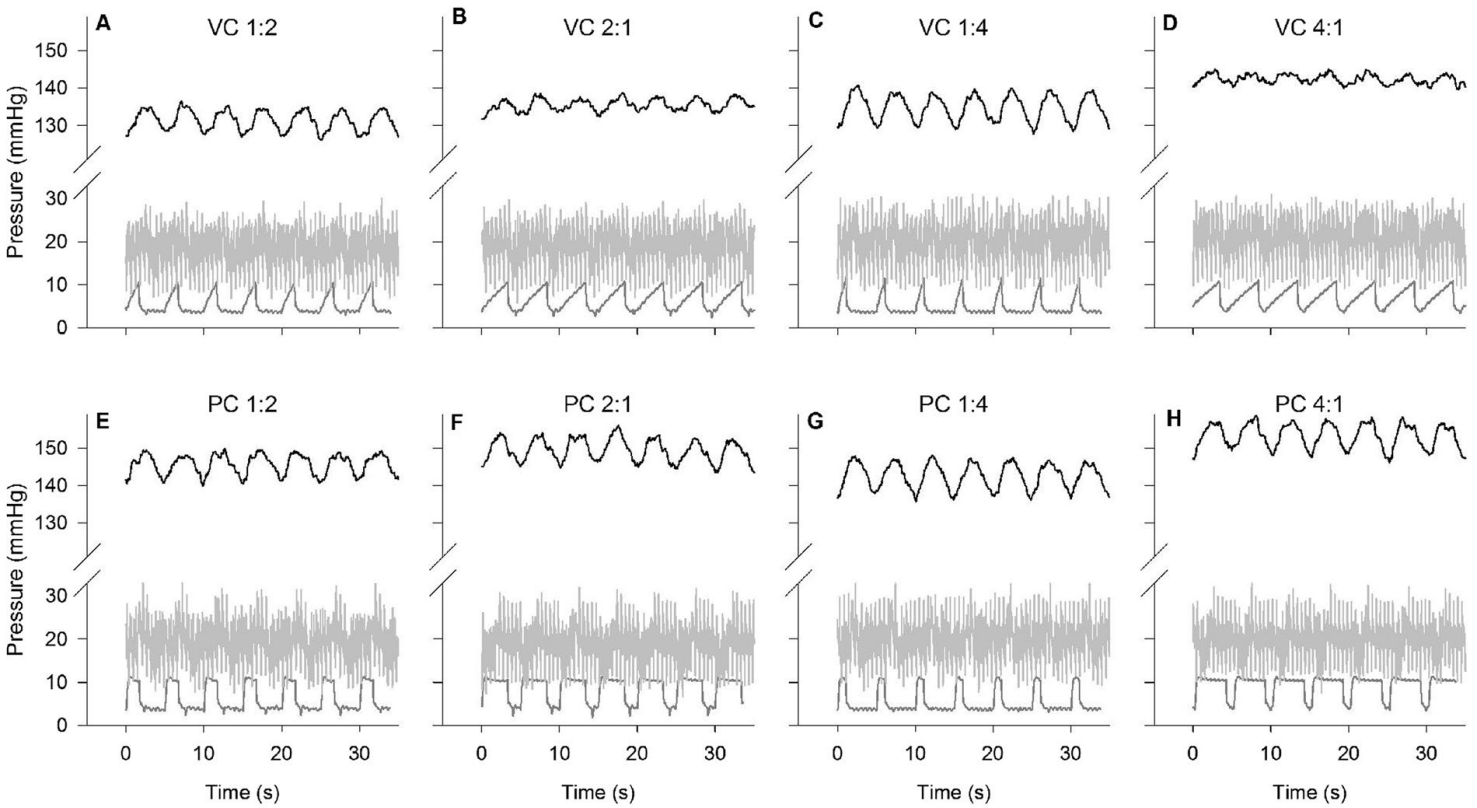
Effects of volume and pressure control ventilation, and inspiration-to-expiration ratio on PaO_2_ oscillations in the uninjured lung at a respiratory rate of 12 breaths per minute. Representative continuous measurements of PaO_2_ (top, black), pulmonary artery pressure (middle, light grey) and airway pressure (bottom, dark grey) are presented as a function of time. Ventilation was managed in volume control (VC, upper panels) or pressure control (PC, lower panels) mode. Inspired-to-expired ratio ranged from 1:4 to 4:1; respiratory rate was 12 breaths per minute. The first set of four experiments started with VC, I:E 1:2, and mean PaO_2_ at 130 mmHg (**A**), followed by a random sequence of PC and inverted ratios (**B**,**E**,**F**) The second set of four experiments started with VC, I:E 1:4, and mean PaO_2_ at 130 mmHg (**C**), followed by a random sequence of PC and inverted ratios (**D**,**G**,**H**).

**Figure 4:**
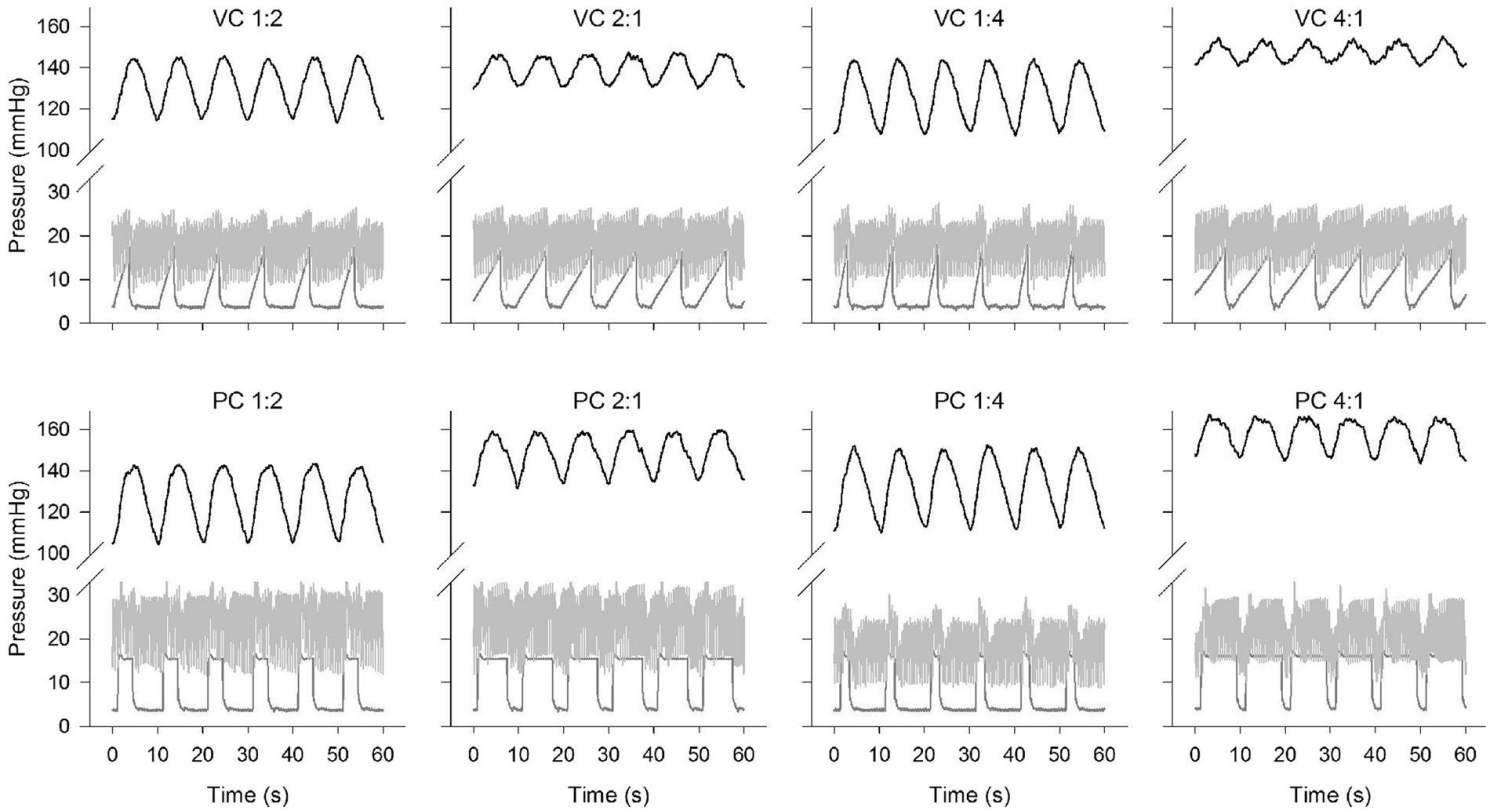
Effects of volume and pressure control ventilation, and inspiration-to-expiration ratio on PaO_2_ oscillations in the uninjured lung at a respiratory rate of 6 breaths per minute. See Fig. 3 legend for details.

Figure 3 shows representative PaO_2_ values over time during volume (A–D) and pressure (E–H) control mechanical ventilation delivered at a RR of 12 breaths per minute, with PEEP set at 5 cmH_2_O. These two ventilation modes and I:E ratios of (A, E) 1:2, (B, F) 2:1, (C, G) 1:4 and (D, H) 4:1 affected both the mean PaO_2_ and the oscillation amplitude. Similarly, Fig. 4 shows representative PaO_2_ values over time for a RR of 6 breaths per minute, where greater changes in mean PaO_2_ levels and larger oscillations were observed.

Table 2 shows the mean PaO_2_ values associated with the eight combinations of mechanical ventilation settings studied at RR of 12 (VT = 10 ml kg^−1^), and the associated mean airway pressure. In the experiments considering I:E of 1:2 and 2:1, the ventilation mode (pressure or volume control) resulted in significant PaO_2_ changes [F(1, 5) = 18.8, *p* = 0.007], as did inverting I:E ratios (i. e. switching the duration of inspiration with that of expiration) [F(1, 5) = 8.4, *p* = 0.03]; no significant interaction effect was observed [*p* > 0.05]. In the experiments considering I:E of 1:4 and 4:1, the ventilation mode resulted in significantly greater PaO_2_ changes [F(1, 5) = 11.6, *p* = 0.02], as did inverting I:E ratios [F(1, 5) = 16.5, *p* = 0.01], with a significant interaction [F(1,5) = 11.5, *p* = 0.02]. The greatest mean PaO_2_ change was observed when I:E was inverted from 1:4 in volume control mode to 4:1 in pressure control mode, when the mean PaO_2_ increased by 19 mmHg, from 125 mmHg (±10 mmHg) to 144 mmHg (±8 mmHg). At RR12 the amplitude of the PaO_2_ oscillations decreased on average from approximately 10 to about 7 mmHg when inverse-ratio ventilation was used. The reduction in amplitude of the PaO_2_ oscillations was particularly clear at RR6 (VT = 20 ml kg^−1^), when inverse-ratio ventilation reduced average PaO_2_ oscillations from 40 to 15 mmHg, observed respectively in pressure control mode (I:E 1:4) and volume control mode (I:E 4:1). Table 3 shows the similar exaggerated changes observed at RR6. At this RR, the greatest mean PaO_2_ change was observed when I:E was inverted from 1:4 in volume control mode to 4:1 in pressure control mode, when mean PaO_2_ increased by 24 mmHg, from 137 mmHg (±18 mmHg) to 161 mmHg (±18 mmHg).

**Table 2:** Mean PaO_2_ value, oscillations amplitudes and mean airway pressure associated with different ventilatory modes (volume or pressure), I:E ratios ranging from 1:4 to 4:1, tidal volume of 10 ml kg^−1^, and respiratory rate of 12 breaths per minute.

I:E	Volume control			Pressure control		
	PaO_2_ mean (mmHg)	PaO_2_ oscillation amplitude (mmHg)	Airway Pressure (cmH_2_O)	PaO_2_ mean (mmHg)	PaO_2_ oscillation amplitude (mmHg)	Airway Pressure (cmH_2_O)
1:2	127 ± 12	10 ± 5	5 ± 2	134 ± 14	10 ± 6	6 ± 3
2:1	127 ± 14	7 ± 4	6 ± 2	140 ± 13	8 ± 4	8 ± 3
1:4	125 ± 10	10 ± 5	5 ± 2	133 ± 9	10 ± 4	5 ± 3
4:1	129 ± 12	5 ± 3	7 ± 2	144 ± 8	7 ± 3	9 ± 2

Values are mean ± standard deviation recorded in 6 animals for 2 min at steady state. RR: respiratory rate. VT: tidal volume.

**Table 3:** Mean PaO_2_ value, oscillations amplitudes and mean airway pressure associated with different ventilatory modes (volume or pressure), I:E ratios ranging from 1:4 to 4:1, tidal volume of 20 ml kg^−1^, and respiratory rate of 6 breaths per minute.

I:E	Volume control			Pressure control		
	PaO_2_ mean (mmHg)	PaO_2_ oscillation amplitude (mmHg)	Airway Pressure (cmH_2_O)	PaO_2_ mean (mmHg)	PaO_2_ oscillation amplitude (mmHg)	Airway Pressure (cmH_2_O)
1:2	133 ± 21^$^	32 ± 9^$^	6 ± 4	113 ± 22*	28 ± 6*	8 ± 5
2:1	146 ± 18^$^	20 ± 7^$^	8 ± 4	127 ± 25*	21 ± 5*	12 ± 5
1:4	137 ± 18*	36 ± 11	5 ± 3	146 ± 15	40 ± 13	6 ± 5
4:1	161 ± 13	15 ± 6	9 ± 4	161 ± 18	22 ± 6	14 ± 5

Values are mean ± standard deviation recorded for 2 min at steady state. RR: respiratory rate. VT: tidal volume. Results from 5 animals are presented, apart from the following *: 6 and $: 7 animals.

### Analysis of CT scans provided no evidence for cyclical atelectasis in the uninjured lung

**Figure 5:**
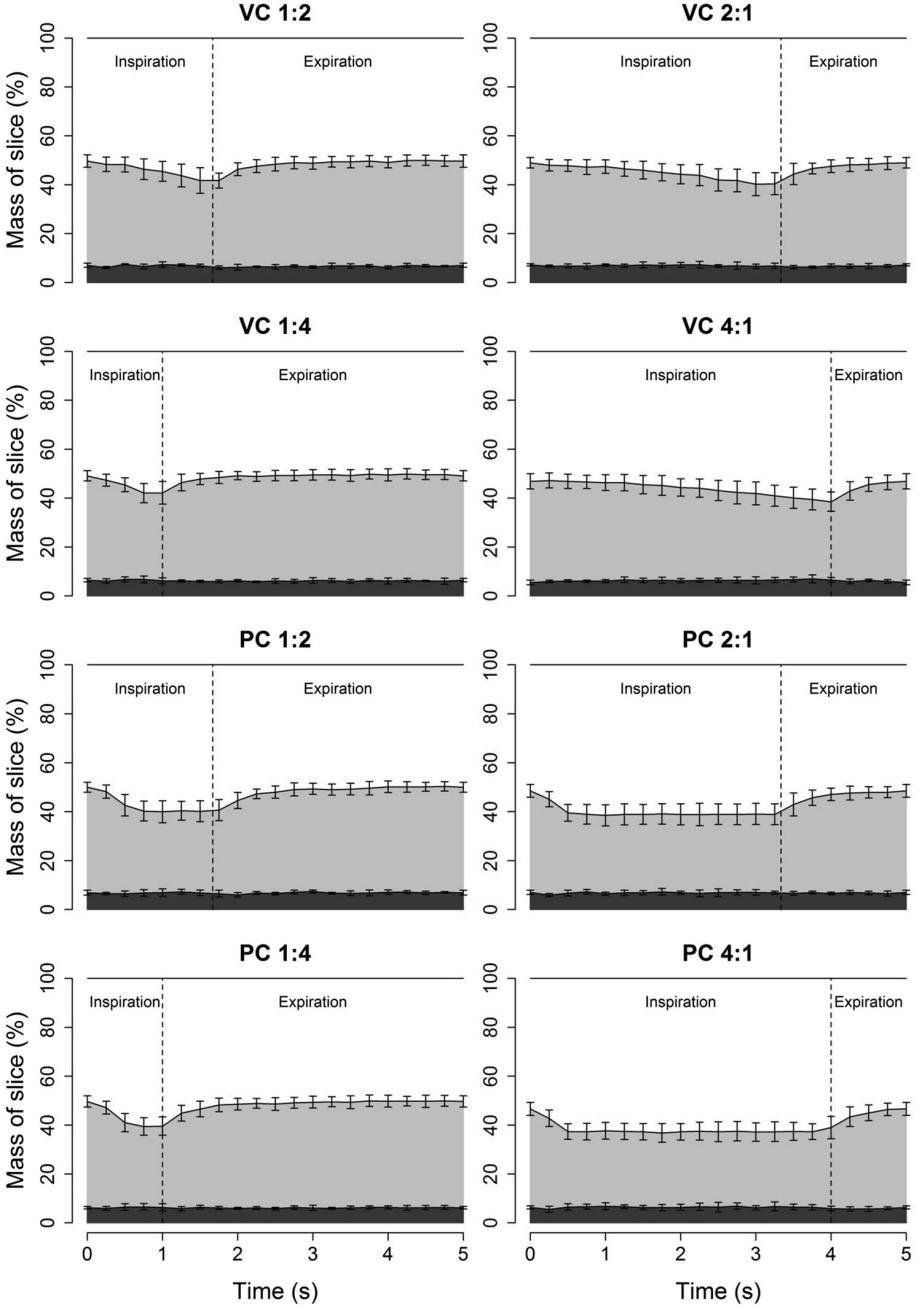
Variation in mass of different density fractions of a single CT slice during tidal ventilation in uninjured animals in different ventilation modes. During inspiration, atelectatic lung (dark grey) decreased marginally with a greater decrease in poorly aerated mass (light grey) and a reciprocal increase in normally aerated lung (white). Overdistended lung represented less than 0.1% of the mass of the slice and was excluded from the figure. Error bars represent SD at each time point. Panel subtitles indicate ventilation mode and I:E ratio. VC – volume control; PC – pressure control.

The change in the mass of the slice that was atelectatic was less than 2% [C.I. 1.5–2.0%] during tidal breathing. Figure 5 shows the proportions of the lung slice mass associated with atelectasis, poor and normal aeration over the period of the respiratory cycle, for each ventilatory condition studied. The atelectatic mass was not different between inspiration and expiration in any of the conditions studied. In contrast, the fraction of normally aerated lung mass increased during inspiration, with a corresponding decrease in the fraction of poorly aerated lung mass. The portion of overinflated lung slice never exceeded 0.1% of the total lung mass.

### A single alveolar compartment model can predict PaO_2_ changes in the uninjured, ventilated lung

**Figure 6:**
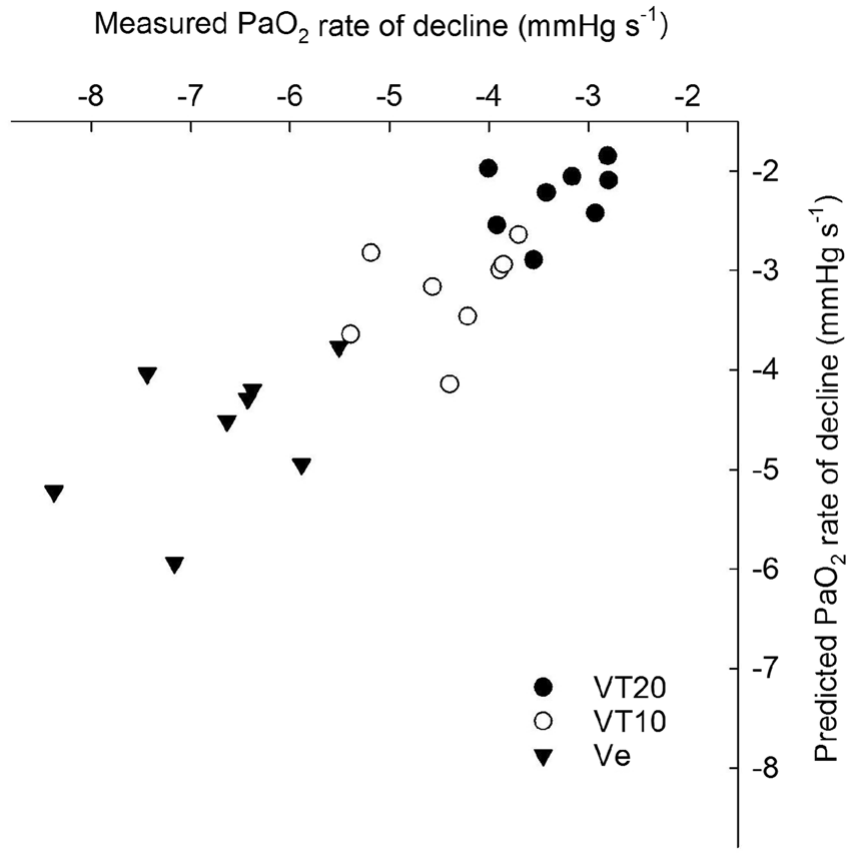
Predicted rate of alveolar PO_2_ decline versus measured rate of PaO_2_ decline during breath hold manoeuvres. Breath hold manoeuvres were performed at end expiratory lung volume (EELV) (Ve, triangles), at the end of an inspiration corresponding to a V_T_ of 10 ml kg^−1^ (VT10, empty circles) and at the end of a large inspiration corresponding to a V_T_ of 20 ml kg^−1^ (VT20, filled circles). Results were obtained in eight animals. EELV was measured in four animals (22.3 ± 3.0 ml kg^−1^), and having found the functional residual capacity (FRC) in ml kg^−1^ in these animals, was used to estimate FRC in the remaining four animals. Alveolar volume was estimated as FRC minus dead space, the latter estimated as 3 ml kg^−1^.

Figure 6 shows the predicted values of ${{\rm{dP}}}_{{\rm{A}}({\rm{t}})}/dt$ according to the proposed model, against the measured values of ${{\rm{dP}}}_{{\rm{A}}({\rm{t}})}/dt$. The predicted values overestimated the measured values by 31%.

**Figure 7:**
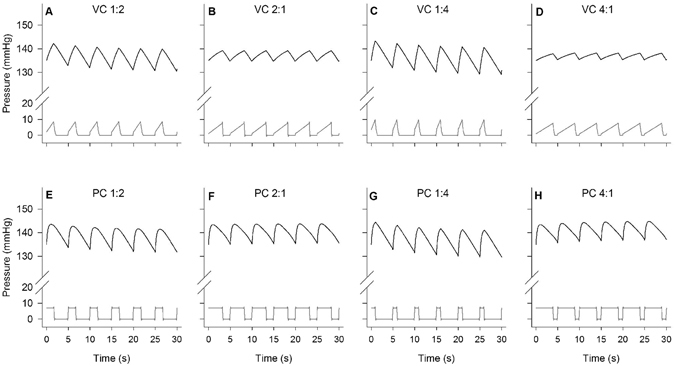
Simulations of PaO_2_ oscillations in the uninjured, ventilated lung from single alveolar model compartment. Simulations of PaO_2_ (top, black) and airway pressure (bottom, dark grey) are presented as a function of time. Ventilation was simulated in volume control (VC, upper panels A,B,C,D) or pressure control (PC, lower panels E,F,G,H) mode. Simulated inspired-to-expired ratio ranged from 1:4 to 4:1, and respiratory rate was 12 breaths per minute.

Figure 7 shows results from the simulations of PaO_2_. These simulations showed changes in mean PaO_2_, and PaO_2_ oscillation amplitudes that were similar to those observed *in vivo* under the various conditions imposed.

## Discussion

In this experimental study in mechanically ventilated pigs with uninjured lungs, we showed that PaO_2_ declined at different rates during breath hold manoeuvres depending on lung volume *in vivo*, that significant dynamic PaO_2_ oscillations occur, and that these PaO_2_ oscillations are determined by alveolar oxygen changes within the respiratory cycle.

During tidal breathing with mechanical ventilation, PaO_2_ oscillations were observed at respiratory rates of 12 and 6 breaths per minute, at mean PaO_2_ values between ~120 and ~150 mmHg, at any I:E ratio and ventilation mode considered in this study. These PaO_2_ oscillations achieved maximum amplitudes greater than 50 mmHg in the absence of cyclical atelectasis as measured by CT image analysis during ventilation in pressure control mode at a RR of 6 breaths per minute, I:E 1:4, VT of 20 ml kg^−1^, and with 5 cmH_2_O PEEP. In this sense, any shunt should be of the same magnitude throughout the respiratory cycle, unless blood flow is redistributed within the breath. The observed increase in PaO_2_ during inspiration suggests that this redistribution of blood flow to non- or poorly ventilated regions did not occur, indicating that it was not a determinant of respiratory PaO_2_ oscillations.

In agreement with the results obtained during breath holds, the weighted-average lung volume was a strong determinant of mean PaO_2_ value, as observed by altering I:E ratios and ventilation control mode. In particular, mean PaO_2_ was higher when inspiration lasted longer than expiration, which is when lung volume is greater across the respiratory cycle. Moreover, the amplitude of the PaO_2_ oscillations was smaller at greater mean lung volume, suggesting that there was a slower PaO_2_ decline during the expiratory phase at I:E of 2:1 and 4:1 than when these I:E ratios were inverted. Overall, these respiratory PaO_2_ oscillations could be predicted from the oscillations in arterial partial pressure of carbon dioxide^24^.

Respiratory PaO_2_ oscillations have previously been interpreted primarily as being caused by cyclical atelectasis associated with tidal recruitment and derecruitment during ventilation in animal models of the acute respiratory distress syndrome (ARDS)^5, 6, 9, 25^. Differences between our work and the published literature include animal species, smaller tidal volumes, greater RR, reduced airway pressures, physiological mean PaO_2_, and crucially that in our work we studied uninjured lung as opposed to animal models of lung injury. It is possible that the use of much larger tidal volumes (as it appears in the literature) might have resulted in larger PaO_2_ oscillations in our uninjured lung model; we did not investigate this possibility as tidal volumes greater than those studied in our experiments are neither physiological, nor of clinical interest^26^. It is intriguing to notice that the PaO_2_ oscillation amplitude of ~22 mmHg observed at I:E of 4:1, RR 6, VT 20 ml kg^−1^, mean PaO_2_ value of ~161 mmHg in our work is similar to that of ~25 mmHg reported in a pig saline lavage model of ARDS in rather similar conditions (I:E 4:1, RR 5, VT 20 ml kg^−1^,mean PaO_2_ value of 425 mmHg)^9, 27^. In this respect, it is possible that the oscillations reported in the literature were caused in part by oxygen mass balance as described in equations 4 and 5 (ventilatory delivery vs pulmonary uptake) rather than cyclical recruitment and derecruitment.

To emphasize the observation that PaO_2_ rate of decline was associated with lung volume, we performed a series of breath hold experiments at EELV with 5 cmH_2_O, end-inspiration and at the end of a large inspiration. We showed that PaO_2_ declined more rapidly at smaller lung volumes than it did at larger lung volumes. This result is in agreement with an original observation performed with slower oxygen saturation sensors during apnea, when the rate of saturation decline was “inversely proportional to the volume of air in the lung during apnea and closely related to the oxygen saturation at the beginning of apnea”^28^.

This difference in the rate of PaO_2_ decline could be partly explained by lung recruitment and derecruitment in anaesthetized animals. Lung recruitment could be observed during end-inspiratory breath holds, especially during a breath hold performed after a large inspiration, and derecruitment could be observed during an end-expiratory breath hold. Lung recruitment during an end-inspiratory breath hold could reduce the rate of PaO_2_ decline, and *vice versa* during an end-expiratory breath hold. However, in this study, we were unable to detect atelectatic mass changes during breath holds using CT image analysis, which did not provide any evidence for recruitment or derecruitment during end-inspiratory or end-expiratory breath holds respectively. Indeed, this result was partly expected given the short duration of the breath hold manoeuvres, the relatively low pressures used during the inspiratory breath holds, the application of 5 cmH_2_O PEEP during the end-expiratory breath holds, and the lack of known lung injury or gross regional ventilation-perfusion mismatch.

Ventilation and perfusion changes during a breath hold could determine real time changes in PaO_2_; in particular, elevated airway pressures applied to the lung during inspiration could cause a prevalence of zone 1 aeration^29, 30^, where the non-dependent lung regions receive little or no perfusion also due to changes in lung blood volume^31^. This phenomenon *per se* would increase the rate of PaO_2_ decline during an end-inspiratory breath hold, due to a reduced oxygen uptake in the zone 1 regions of the pulmonary circulation. The highest airway pressure applied in our experiments was 24 cmH_2_O during breath holds at the end of a large inspiration, when the rate of PaO2 decline was slower than during end-inspiratory and end-expiratory breath holds. Importantly, the airway pressure did not exceed the pulmonary artery pressure at any time during breath holds, and overinflated lung mass was never greater than 0.1%, suggesting that zones 1 were not dominant, hence did not affect the rate of PaO_2_ decline.

Two main limitations of this technology are that it is unable to demonstrate whether a respiratory PaO_2_ change is caused by alterations in either ventilation, or perfusion, or both, and that it does not provide evidence for their spatial distribution within the lung. In our study, the dynamic CT image analysis partly compensated for this limitation by providing an index of lung aeration and atelectasis. Although this information was anatomically limited to a single slice during tidal breathing, the antero-posterior orientation of the slice could be representative for the effect of the gravitational gradient on pulmonary aeration^32^.

Our study demonstrates that cyclical atelectasis is not necessary for respiratory PaO_2_ oscillations to appear during mechanical ventilation. We conclude that the mechanism determining these respiratory PaO_2_ oscillations is the variation of alveolar oxygen tension within the breath, with oxygen entering the alveoli during each inspiration, and that these cyclic variations in alveolar oxygen tension are transmitted all the way to the systemic arteries. Our results in an anaesthetized, ventilated pig model, in the absence of lung injury, may have implications for the interpretation of the results from conditions of lung disease^33^, in particular from patients with lung injury, and from animal models of ARDS.

## Material and Methods

The study was approved by the Animal Research Ethics Committee at Uppsala University and by the UK Home Office. All the animal experiments conformed to the National Institutes of Health Guidelines for the Use of Laboratory Animals. Relevant sections of the ARRIVE guidelines were followed. A total of 8 domestic pigs were studied (average ± SD: 29 ± 2 kg). Details regarding the anaesthesia, mechanical ventilation, instrumentation and measurements are reported in the supplementary information.

### Continuous oxygen sensing

The intravascular, fibre optic oxygen sensor was inserted in a carotid artery via a standard arterial catheter, and PaO_2_ was recorded continuously, simultaneously with analogue signals for cardiovascular and respiratory parameters from patient monitors.

### Experimental protocol

#### Breath hold manoeuvres

In order to determine the rate of PaO_2_ decline during apnoea, breath hold manoeuvres lasting at least 20 s were performed at three lung volumes: end-expiration (end-expiratory lung volume, EELV, defined as functional residual capacity with 5 cmH_2_O PEEP), end of an inspiration corresponding to a V_T_ of 10 ml kg^−1^ (VT10), and at the end of a large inspiration, corresponding to a V_T_ of 20 ml kg^−1^ (VT20). Breath hold manoeuvres associated with these three lung volumes were performed six times each, in a sequence that covered each possible sequential effect, for a total of 18 breath hold manoeuvres per animal. The sequence in which breath hold manoeuvres were performed is presented in Table S1. PaO_2_ decline was normalized to account for small differences in body weight between animals. The steepest rate of decline measured continuously over 5 s during each breath hold manoeuvre was considered for analysis. This period of 5 s was used to maximize the interval time while avoiding the inclusion of PaO_2_ values below 100 mmHg, when the rate of PaO_2_ decline decreases due to haemoglobin desaturation, which is beyond the scope of our study.

#### Tidal breathing

We aimed to establish the effect of altering I:E ratios (1:2 vs 2:1, and 1:4 vs 4:1) on mean PaO_2_ and PaO_2_ oscillation amplitudes, and the potential effect of cyclical atelectasis, if present, in determining PaO_2_ during tidal breathing. We studied the effects of ventilation in pressure control mode, and also explored the effect of delivering the same V_T_ gradually in volume control mode, recording PaO_2_ changes and determining oscillation amplitudes at the four I:E ratios. Airway pressure was monitored and controlled throughout the experiments. The two sets of tests started with either I:E of 1:2 or 1:4 in volume control mode achieving a mean PaO_2_ of about 130 mmHg, followed by a randomised sequence of any possible combination of I:E and pressure or volume control mode. Respiratory rates (RR) studied included 12 and 6 breaths per minute, with V_T_ of 10 ml kg^−1^ and 20 ml kg^−1^ respectively. In total, we included 16 conditions (four I:E ratios, each in two control modes, and at two respiratory rates). During tidal breathing, once steady state had been achieved, data collected over a period of 2 min were considered for analysis. Steady state was defined as mean PaO_2_ not changing by more than 5 mmHg min^−1^.

PEEP of 5 cmH_2_O was used throughout the experiments in order to prevent, or at least reduce the likelihood of collapse of dependent regions at end expiration.

### Computed tomography imaging

We used computed tomography (CT) to measure lung volumes as well as to estimate pulmonary atelectasis and its changes during the breath hold manoeuvres and dynamically during tidal breathing in two animals. Details of the CT images acquisition, analysis procedure, calculation of air volume and tissue mass are presented in the supplementary information.

### A model of the uninjured porcine lung

We hypothesised that the uninjured lung could be modelled as a single alveolar compartment with a constant oxygen uptake. The rate of change of oxygen in this compartment is equal to the difference in the rates of uptake by the pulmonary circulation and input via ventilation during inspiration, or elimination during expiration. Details of the mathematical modelling are presented in the supplementary information.

### Statistical analysis

PaO_2_ data recorded during the breath hold manoeuvres were analysed by means of univariate repeated measures ANOVA with lung volumes as fixed factors (IBM SPSS Statistics, Version 22.0; Armonk, NY, USA), and post hoc analysis with the Bonferroni method. A two-way ANOVA was conducted to compare the main effects of ventilation mode and I:E ratio, and their interaction on mean PaO_2_ and its oscillations. Ventilation mode included two levels (volume and pressure control mode), and I:E ratio consisted of two levels (either 1:2 and 2:1, or 1:4 and 4:1). Values presented are mean ± standard deviation, unless otherwise stated. Significance level was accepted at *p* < 0.05.

### Data availability statement

Data are available upon request.

## Electronic supplementary material

Supplementary Information
