# Supplementary material for: Respiratory oscillations in alveolar oxygen tension measured in arterial blood
Source: Sci Rep. 2017 Sep 6;7:7499. doi: 10.1038/s41598-017-06975-6 (PMC5587703; doi:10.1038/s41598-017-06975-6)
Supplement: Supplementary file 1 — Supplementary Information [file 41598_2017_6975_MOESM1_ESM.pdf]

Respiratory oscillations in alveolar oxygen tension measured in arterial blood

Federico Formenti, Nikhil Bommakanti, Rongsheng Chen, John N. Cronin, Hanne McPeak, Delphine Holopherne-Doran, Goran Hedenstierna, Clive E. W. Hahn, Anders Larsson, Andrew Farmery

Supplementary Information

SI Materials and Methods

Studies performed in the Hedenstierna laboratory were approved by the animal ethics committee at the University of Uppsala, and those performed at the School of Veterinary Sciences, University of Bristol by the UK Home Office.

Anaesthesia, instrumentation and measurements

The animals, lying in dorsal recumbency, received general IV anaesthesia using a combination of ketamine, fentanyl, and midazolam. Adequacy of anaesthesia was established by the absence of movement, haemodynamic monitoring (heart rate and arterial pressure), and absence of reflexes. Once anaesthesia was ascertained, pancuronium was administered for muscle relaxation. Continuous infusion of Ringerfundin^{TM} solution (Braun Melsungen Ag, Melsungen, Germany) was used as a fluid replacement in doses of 10 ml kg^{-1} h^{-1} during the preparation and reduced to 7 ml kg^{-1} h^{-1} thereafter.

Baseline ventilation was delivered in pressure-controlled mode (Servo-I, Maquet, Solna, Sweden) with PEEP set at 3.7 mmHg (5 cm H_2O), and a pressure above PEEP sufficient to deliver a tidal volume (V_T) of 8 mL kg^{-1}. Respiratory rate (RR) was adjusted to keep $PaCO_2$ between 35 and 45 mmHg, inspiratory-to-expiratory ratio (I:E) at 1:1, and F_IO_2 was adjusted to maintain normoxemia. A catheter was placed in a peripheral artery for blood pressure measurement. Pulmonary artery pressure was measured by means of a Swan-Ganz catheter, and ECG, arterial pressure, SpO_2 and end-tidal carbon dioxide were monitored throughout. Cardiac output was monitored intermittently by thermodilution. In one experiment, cardiac output was measured in triplicates during the breath hold manoeuvres performed at end expiration, end of an inspiration corresponding to a V_T of 10 ml kg^{-1} (VT10), and at the end of a large inspiration corresponding to a V_T of 20 ml kg^{-1} (VT20).

Baseline physiological variables were continuously monitored with standard patient monitors (Datex Ohmeda Capnomac Ultima; multi-parameter patient monitor: Datex AS3). Analogue signals were continuously recorded on a computer via PowerLab (AD Instruments, New Zealand).

Sequence of breath hold manoeuvres

The sequence in which breath hold manoeuvres were performed is presented in Table S1. This sequence was chosen in order for each manoeuver to be preceded and followed by the other two manoeuvres, to take into account for any potential confounding sequential effects.

1	Ve
2	VT10
3	VT20
4	Ve
5	VT20
6	VT10
7	VT20
8	Ve
9	VT10
10	VT20
11	VT10
12	Ve
13	VT10
14	Ve
15	VT20
16	VT10
17	VT20
18	Ve

Table S1. Sequence of breath hold manoeuvres performed at (Ve) end-expiration [functional residual capacity, FRC, plus PEEP set at 3.7 mmHg (5 cmH_2O)], (VT10) end of an inspiration corresponding to a V_T of 10 ml kg^{-1}, and (VT20) at the end of a large inspiration corresponding to a V_T of 20 ml kg^{-1}.

Computed tomography imaging and analysis

CT images were acquired with a Somatom Definition Flash (Siemens, Erlangen, Germany). CT voxel dimensions were 5 x 0.5 x 0.5 mm. In order to determine the degree of collapse or recruitment (potential determinants of PaO_2) during the breath hold manoeuvres, two whole lung scans were performed: one as soon as the breath

hold manoeuvre began, and one just before tidal breathing was restored. We also performed dynamic CT scanning from a single juxtadiaphragmatic slice at a temporal resolution of 50 ms (i. e. 20 images per second) during tidal ventilation in each ventilatory condition in order to determine the degree of cyclical atelectasis, a potential determinant of PaO_2 and its respiratory oscillations.

The breath-hold images were collected with 80 kV tube voltage, 364 mA tube current and 64 x 0.6 mm collimation. During tidal breathing, images of a single juxtadiaphragmatic slice were collected at 50 ms intervals with 70 kV tube voltage, 246 mA tube current, 32 x 1.2 mm collimation. The juxtadiaphragmatic slice location was chosen because changes seen here have been reported to closely approximate those within the entire lung (1). Reconstituted voxel size was 0.5 x 0.5 x 5 mm in all cases.

CT image analysis was performed with 3D Slicer version 4.5.0-1 (2). Lung parenchyma was manually segmented on a slice-by-slice basis with 3D Slicer version 4.5.0-1 (2). Diaphragm, mediastinum, hilar vessels and inferior vena cava were excluded from the analysis. Other intra-pulmonary vessels and airways and trachea up to the level of the clavicles were included. The rationale for this was that even when it was possible to completely identify vessels and airways down to the voxel level (i.e. the limit of delineation), the branching level at which small vessels and airways became smaller than a voxel was likely to vary during the respiratory cycle due to variation in airway pressures; by including them all this variation was

eliminated with the acknowledgement that a small, but constant, error was added to all images.

Calculation of voxel count and mean density was performed using a bespoke tool over the entire lung (or slice for dynamic series). The lung was automatically segmented into four regions based on density: atelectatic lung (-100 to +100 Hounsfield Units (HU)), poorly aerated lung (-500 to -101 HU), normally aerated lung (-900 to -501 HU) and overdistended lung (-1000 to -901 HU) as per Gattinoni *et al.*(3), Lundquist *et al.* (4) and Vieira *et al.* (5). CT images were recorded at 20 Hz; every fifth image was considered for analysis, giving a final temporal resolution of 250 ms.

Calculation of air volume and tissue mass

By assuming the lung to be composed of two separate components, namely water with a density of 1 g cm^{-3} (0 HU), and air with a density of 0 g cm^{-3} (-1000 HU), it was possible to calculate the air volume and tissue mass within a single slice using the following equations:

$$Air\,Volume = Zone\,Area \times Slice\,Thickness \times \frac{-CT\,Density}{1000}$$

$$Tissue\,Mass = Zone\,Area \times Slice\,Thickness \times \left(1 + \frac{CT\,Density}{1000}\right)$$

The fractional mass of each zone was calculated as the ratio of the mass of that zone to the mass of the entire lung (or slice in case of the tidal ventilation scans).

Tidal volume measured by CT was calculated for each breath-hold manoeuvre by first identifying the mean air volume of the whole lung at the start of the end-expiration breath-hold for each animal, and then subtracting that volume from the individual whole lung air volumes for each of the end-inspiration and end-large inspiration breath-hold manoeuvres.

In one of the two animals studied with CT, functional residual capacity (FRC) with PEEP set at 3.7 mmHg (5 cmH_2O) was also confirmed by means of sulfur hexafluoride washout, as presented elsewhere (6).

A model of the uninjured porcine lung

The oxygen in the alveolar compartment at any time equals $V_{A(t)}F_{A(t)}$, where $V_{A(t)}$ is the alveolar gas volume and $F_{A(t)}$ is the alveolar oxygen gas fraction at time t.

The associated rate of change of oxygen in the alveolar compartment is:

$$d\frac{(V_{A(t)}F_{A(t)})}{dt} = V_{A(t)}\frac{dF_{A(t)}}{dt} + F_{A(t)}\frac{dV_{A(t)}}{dt} \qquad (1)$$

In inspiration, the difference between the rate of oxygen intake via ventilation, and removal by the pulmonary circulation ($\dot{v}_{O2}$) equals

$$\frac{dV_{A(t)}}{dt}F_{IO2} - \dot{v}_{O2} \qquad (2)$$

For expiration, this equals

$$-\frac{dV_{A(t)}}{dt}F_{A(t)} - \dot{V}_{O2} \quad (3)$$

Mass balance equations can be obtained by combining equations 2 and 1 (for inspiration) and 3 and 1 (for expiration):

Inspiration: $$\frac{dF_{A(t)}}{dt} = \frac{\dot{V}_{O2} + \frac{dV_{A(t)}}{dt}(F_I - F_{A(t)})}{V_{A(t)}} = \frac{\dot{V}_{O2} + \dot{V}_{insp_{(t)}}(F_I - F_{A(t)})}{EELV + V_{insp_{(t)}}} \quad (4)$$

Expiration: $$\frac{dF_{A(t)}}{dt} = \frac{\dot{V}_{O2}}{V_A} = \frac{\dot{V}_{O2}}{EELV + V_T - V_{exp_{(t)}}} \quad (5)$$

where $\dot{V}_{insp_{(t)}}$ is the inspiratory flow rate, EELV is end-expiratory lung volume, and $V_{insp_{(t)}}$ and $V_{exp_{(t)}}$ are the time variant inspired and expired volumes respectively.

The terms F_A and dF_A/dt can be converted to partial pressures by multiplying them by the barometric pressure, and in uninjured lung, alveolar and arterial partial pressure are nearly equal.

Solutions to equations 4 and 5 were used to plot simulations of F_AO_2, which we assumed to be representative of PaO_2, in the presence of a constant alveolar to arterial gradient.

During breath-holds, the rate of decline of PaO_2 was given by a modification to equation 5:

$$\frac{dF_{A(t)}}{dt} = \frac{\dot{V}_{O2}}{EELV + V_T} \quad (6)$$

where at end expiration VT = 0, and at end inspiration VT = 10 or 20 ml kg^{-1} for normal or large breaths respectively.

Using this model, predicted values of $\mathrm{dF}_{\mathrm{A(t)}}/dt$ can be calculated for each animal for each of the lung volumes considered during the breath hold manoeuvre experiments, from measured values of $\dot{\mathrm{v}}_{\mathrm{O2}}$ and EELV.

At the end of each non-recovery study, the animals were killed under anaesthesia with an overdose of pentobarbital (100 mg kg^{-1}).

SI Results

Figure S1. Agreement between V_T measured via airway flow and CT imaging.

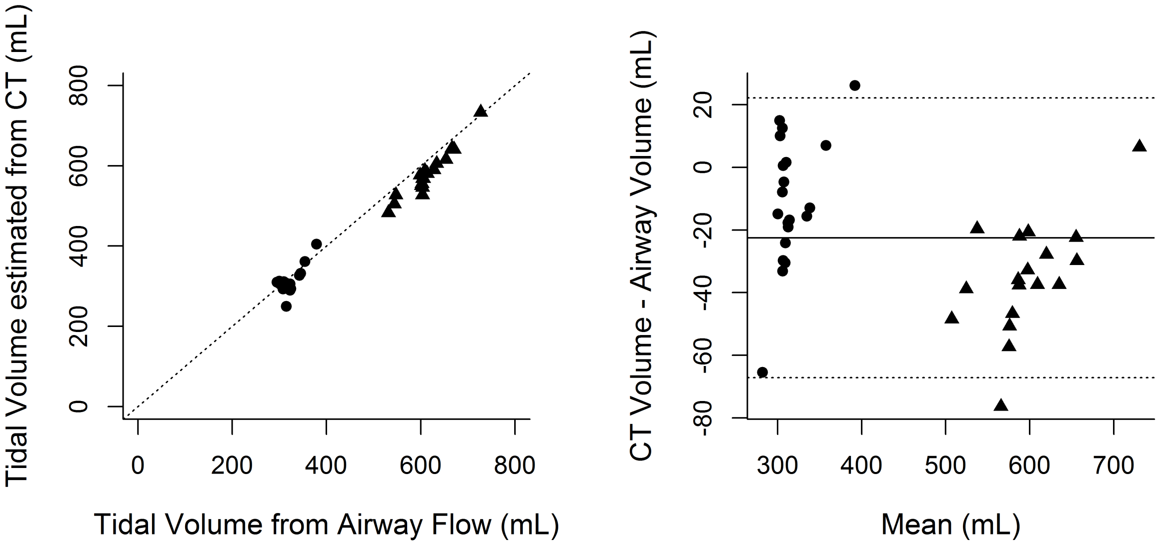

Data are from breath holds performed at end-inspiration (circles) and end-large inspiration (triangles). Left: The distribution of measured values is due to different size animals and to some measurements having been obtained during pressure control ventilation, where the actual V_T was an estimate of either 10 ml Kg^{-1} or 20 ml Kg^{-1}. The dotted line is the identity line. Right: Bland-Altman analysis of the agreement between V_T measured via airway flow and CT imaging. Solid line is mean difference and dotted lines 95% limits of agreement (±1.96 SD), which include the zero difference value.

Figure S2. Representative CT images and their segmentation for breath hold manoeuvres.

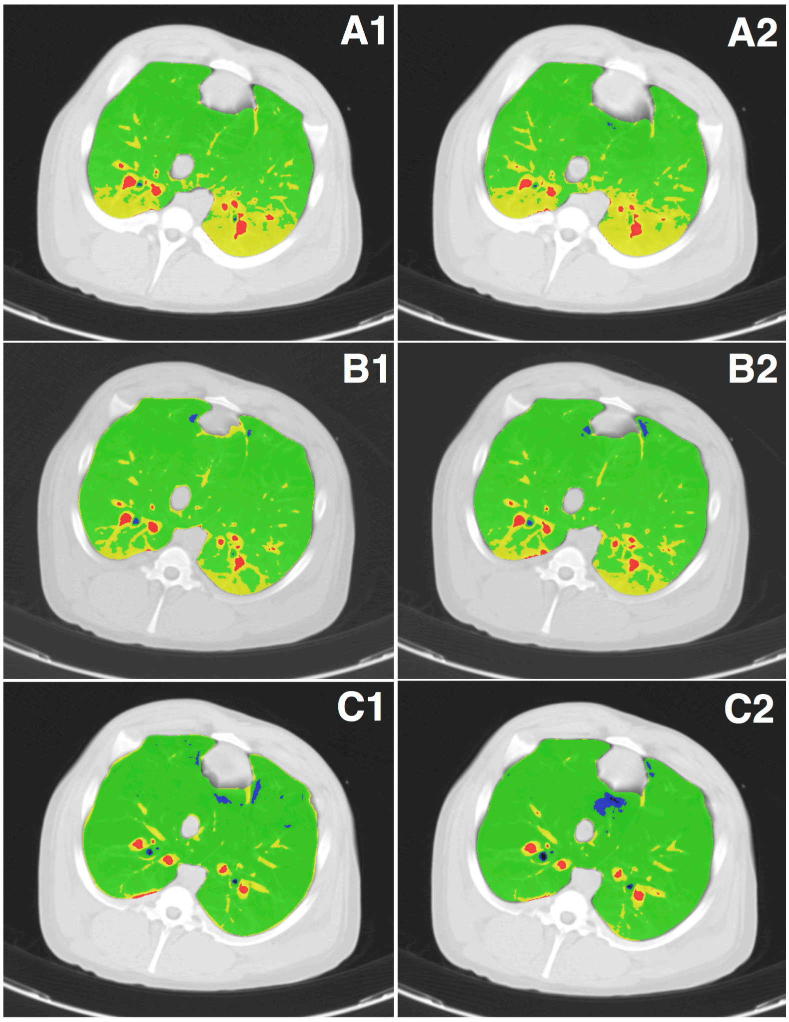

Single slice CT scans and corresponding superimposed segmentation recorded (A1, B1, C1) as soon as a breath hold manoeuvre began, and (A2, B2, C2) just before tidal breathing was restored at (A1 and A2) end-expiratory lung volume with positive end-expiratory pressure of 5 cmH_2O, (B1 and B2) for an inspiration corresponding to VT 10 ml kg^{-1}, and (C1 and C2) for a large inspiration corresponding to VT 20 ml kg^{-1}. Each panel shows slice segmentation, where green is normally aerated (-900 to -501 HU), yellow is poorly aerated (-500 to -101 HU), and red is atelectasis or pulmonary vessels (-100 to +100 HU).

Figure S3. Representative three-dimensional reconstruction of the lung from CT images for breath hold manoeuvres.

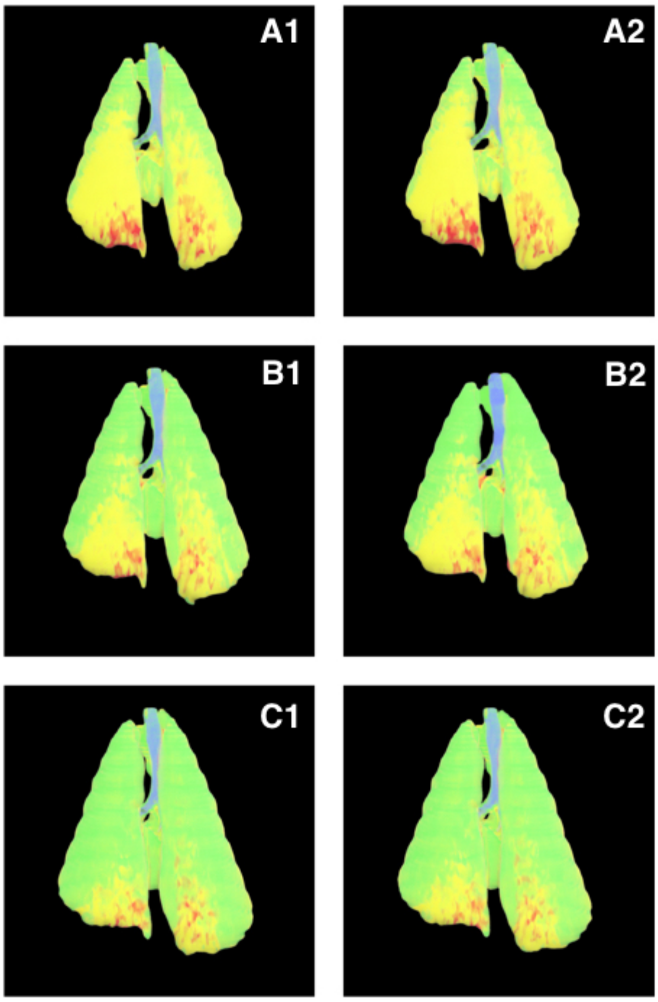

Reconstructions of the lung were composed from whole lung CT scans recorded (A1, B1, C1) as soon as a breath hold manoeuvre began, and (A2, B2, C2) just before tidal breathing was restored at (A1 and A2) end-expiratory lung volume with positive end-expiratory pressure of 5 cmH_2O, (B1 and B2) for an inspiration corresponding to VT 10 ml kg^{-1}, and (C1 and C2) for a large inspiration corresponding to VT 20 ml kg^{-1}. Each panel shows slice segmentation, where green is normally aerated (-900 to -501 HU), yellow is poorly aerated (-500 to -101 HU), and red is atelectasis (-100 to +100 HU).

Cardiac output varied between the three lung volumes tested in the experiments where it was recorded. Cardiac output ranged from 3.8 ± 0.2 $l\ min^{-1}$ during the breath hold manoeuvre at end-expiration to 2.9 ± 0.1 $l\ min^{-1}$ at the end of a large inspiration (VT20) (Table S2).

Table S2. Cardiac output recorded by thermodilution during breath hold manoeuvres.

**Lung volume (l)**	**Cardiac output ($l\ min^{-1}$)**
FRC + 5cm$H_2$0	3.8 ± 0.2
V_T10	3.5 ± 0.3
V_T20	2.9 ± 0.1

Cardiac output decreased when breath hold manoeuvres were performed at elevated inspiratory airway pressures (V_T10 and V_T20). Values are mean ± SD (n = 3 per condition).

SI Discussion:

It is possible that greater mean PaO_2 values are observed during the I:E 4:1 condition in association with auto-PEEP (i.e. increased EELV). This phenomenon is observed when insufficient time is allowed for the lung to return to its original EELV, in which case the EELV can, up to a point, increase over time. The most challenging

scenario considered in our study in this respect was the condition where a RR of 12 breaths per minute and I:E of 4:1 was studied, when the duration of the expiratory period was 1 s, a period similar to that observed in spontaneous breathing in the pig. Like in spontaneous breathing, expiration was passive during our experiments and we measured no expiratory flow at the end of expiration, so we do not expect auto-PEEP to have occurred even in the most challenging condition studied.

Elevated airway pressures can reduce cardiac output and limit pulmonary perfusion during end-inspiratory breath holds, resulting in a reduced PaO_2. Although the high airway pressures used in our experiments did not exceed 24 cmH_2O, we explored the possibility that cardiac output could be limited during breath holds in one animal, recording triplicate cardiac output measurements by standard thermodilution. Cardiac output was 3.8 ± 0.2 l min^{-1} during the end-expiratory breath holds, 3.5 ± 0.3 l min^{-1} during the end-inspiratory breath holds, and was reduced to 2.9 ± 0.1 l min^{-1} for breath holds performed at the end of a large inspiration. This reduction was associated with mild arterial blood pressure reduction, and no measurable change in heart rate. Despite cardiac output decreasing with increased airway pressures applied during the breath holds, its minimum measured value appeared sufficient to maintain pulmonary perfusion pressure and was unlikely a determinant of PaO_2 changes.

Arterial oxygen saturation was maintained at or above 99% throughout the experiments because lower haemoglobin saturation would have affected the dynamic PaO_2 changes observed within breath. Having minimized the potential effect of arterial oxygen saturation changes, we conclude that dynamic PaO_2 oscillations are

determined by alterations in alveolar oxygen mass balance within a respiratory cycle in the uninjured lung of the anaesthetized pig.

SI References:

1. Lu Q, Malbouisson LM, Mourgeon E, Goldstein I, Coriat P, Rouby JJ. Assessment of PEEP-induced reopening of collapsed lung regions in acute lung injury: are one or three CT sections representative of the entire lung? *Intensive Care Med* 2001; 27: 1504-1510.

2. Fedorov A, Beichel R, Kalpathy-Cramer J, Finet J, Fillion-Robin J-C, Pujol S, Bauer C, Jennings D, Fennessy F, Sonka M. 3D Slicer as an image computing platform for the Quantitative Imaging Network. *Magnetic resonance imaging* 2012; 30: 1323-1341.

3. Gattinoni L, Pesenti A, Bombino M, Baglioni S, Rivolta M, Rossi F, Rossi G, Fumagalli R, Marcolin R, Mascheroni D, et al. Relationships between lung computed tomographic density, gas exchange, and PEEP in acute respiratory failure. *Anesthesiology* 1988; 69: 824-832.

4. Lundquist H, Hedenstierna G, Strandberg A, Tokics L, Brismar B. CT-assessment of dependent lung densities in man during general anaesthesia. *Acta Radiol* 1995; 36: 626-632.

5. Vieira SR, Puybasset L, Richecoeur J, Lu Q, Cluzel P, Gusman PB, Coriat P, Rouby JJ. A lung computed tomographic assessment of positive end-

expiratory pressure-induced lung overdistension. *Am J Respir Crit Care Med* 1998; 158: 1571-1577.

6. Larsson A, Linnarsson D, Jonmarker C, Jonson B, Larsson H, Werner O. Measurement of lung volume by sulfur hexafluoride washout during spontaneous and controlled ventilation: further development of a method. *Anesthesiology* 1987; 67: 543-550.
